# Of scents and cytokines: How olfactory and food aversions relate to nausea and immunomodulation in early pregnancy

**DOI:** 10.1093/emph/eoaf016

**Published:** 2025-09-24

**Authors:** Dayoon Kwon, Daniel M T Fessler, Delaney A Knorr, Kyle S Wiley, Julie Sartori, David A Coall, Molly M Fox

**Affiliations:** Department of Epidemiology, University of California, Los Angeles, Los Angeles, CA, USA; Department of Anthropology, University of California, Los Angeles, Los Angeles, CA, USA; Center for Behavior, Evolution, & Culture, University of California, Los Angeles, Los Angeles, CA, USA; Bedari Kindness Institute, University of California, Los Angeles, Los Angeles, CA, USA; Department of Evolutionary Anthropology, Duke University, Durham, NC, USA; Department of Sociology and Anthropology, University of Texas at El Paso, El Paso, TX, USA; School of Medical and Health Sciences, Edith Cowan University, Joondalup, Australia; School of Medical and Health Sciences, Edith Cowan University, Joondalup, Australia; Department of Anthropology, University of California, Los Angeles, Los Angeles, CA, USA; Department of Psychiatry and Biobehavioral Sciences, University of California, Los Angeles, Los Angeles, CA, USA

**Keywords:** pregnancy, aversions, immunomodulation, immune response, inflammation, morning sickness

## Abstract

**Background:**

During pregnancy, the maternal body undergoes extensive physiological adaptations to support embryonic growth, including whole-body remodeling, that may induce odor and food aversions, as well as nausea and vomiting. The biological mechanisms behind odor and food aversions, as well as nausea and vomiting in early pregnancy, remain largely unexplored. Our study investigated associations between these changes and cytokine profiles during pregnancy.

**Methodology:**

A cohort of pregnant Latina women in Southern California (*n* = 58) completed a structured questionnaire on pregnancy “morning sickness”-related symptoms and aversions. Maternal plasma cytokine levels were measured between 5 and 17 weeks’ gestation.

**Results:**

About 64% of participants experienced odor or food aversions, primarily to tobacco smoke and meat; 67% reported nausea, and 66% experienced vomiting. Multivariable linear regression models revealed that odor aversions were associated with increased pro-inflammatory T-helper-cell type (Th) 1 composite cytokine levels. Women who found tobacco smoke aversive exhibited a shift toward Th1 immune responses, indicated by a higher Th1:Th2 ratio. Food aversions also showed a positive association with Th1 cytokine levels. A borderline positive association was noted between nausea and vomiting and the Th1:Th2 ratio.

**Conclusions:**

These findings are consistent with the hypothesis that gestational changes in olfactory and gustatory experience, and nausea and vomiting, reflect adaptive upregulation of behavioral prophylaxis in ways that could protect the fetus. If this elevated Th1:Th2 ratio and pro-inflammatory phenotype are part of the maternal and embryonic response to embryogenesis, the behavioral and biological markers that we explore may provide an accessible index of fetal development during early pregnancy.

## INTRODUCTION

During early pregnancy, it is common for women to develop aversions to certain odors and foods and to experience nausea and vomiting. However, the biological mechanisms underlying these phenomena are incompletely understood. Given their postulated adaptive functions and potential clinical significance, it is important to explore these biomechanisms. While immune alterations during pregnancy have been studied, connections between immunological changes, aversions, and nausea and vomiting during pregnancy (NVP) are not well established. To illuminate the biological processes involved, we examined the interplay between cytokines, olfactory experience, and behavioral responses during early pregnancy. Each stage of pregnancy has a unique immunological profile; we focus on indexical cytokines in early-to-mid pregnancy, as this is when aversions, nausea, and vomiting primarily occur.

**Figure 1:**
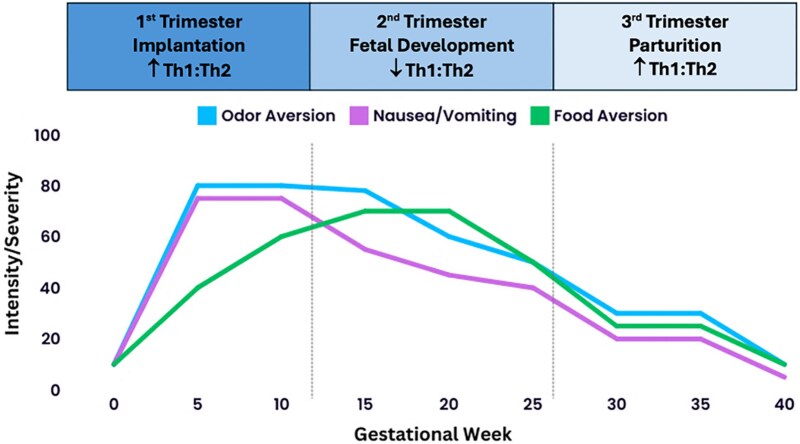
A conceptual model of the behavioral and immunological changes during pregnancy. Note: The intensity/sensitivity levels depicted are not based on specific observational data but are provided to facilitate a structured understanding of the phenomena under investigation, grounded in empirical evidence [6, 7].

The first and early second trimesters involve an inflammatory response for successful implantation and placentation [1]. Implantation is an invasive process in which the blastocyst breaks through the epithelial lining of the uterus, restructures the endometrial tissue, and connects to the maternal blood supply [1]. This invasion and restructuring is met by a maternal response characterized by a T-helper-cell type 1 (Th1) dominant or pro-inflammatory phase [2], similar to the inflammatory response to parasitic infection [3]. During this pro-inflammatory phase, an elevated Th1:Th2 ratio is part of the body’s normative immune response, necessary for the conceptus’ survival and adaptation to the maternal environment (Fig. 1). In principle, increased production of Th1 pro-inflammatory cytokines could contribute to aversions and nausea, as these can affect the central nervous system. The nausea and emetic reflex, situated below the blood–brain barrier, is susceptible to changes in circulating proteins and signaling factors in maternal blood and tissue fluid [4]. Heightened nausea during early pregnancy may thus result from the surge in pro-inflammatory cytokines. Although higher levels of pro-inflammatory cytokines are observed during the first trimester, implantation involves a complex interplay of immune processes rather than a straightforward inflammatory response [5].

The second trimester is characterized by the suppression of the pro-inflammatory Th1 immune state, activation of an anti-inflammatory Th2-dominant state, and a decrease in circulating natural killer cells and IFN-γ, followed by an increase in Th1 cells in the third trimester [8]. These immunological changes are intricately connected to establishing a tolerogenic environment for the fetus early in pregnancy. Given the possibility that NVP, as well as odor and food aversions, may serve as part of the behavioral immune system [9], potentially protecting women from consuming risky foods [10], we explore whether there is a connection between immunomodulatory mechanisms and subjective symptom experience.

About 50%–85% of women experience food aversions during the first half of pregnancy [11–13]. The most common targets of gestational food aversions are meat, fish, caffeinated beverages (principally coffee and tea), and various spices [13, 14]. Although rates vary across populations, up to 80% of pregnant women experience nausea, and over 50% suffer vomiting [15, 16]. NVP typically begins early in the first trimester, generally subsiding by the end of the second trimester [17]. Although hyperemesis gravidarum (severe nausea and vomiting, a complication of pregnancy) endangers both mother and fetus [18], modest levels of NVP correlate with positive birth outcomes, particularly when it occurs early in pregnancy (see Emmott [19], for review). If immunomodulatory mechanisms indeed underlie NVP, then the continuum from moderate NVP to hyperemesis gravidarum may reflect differences in degree rather than kind (e.g. pathological NVP may reflect an excessive Th1 response). Mechanisms underlying gestational food aversions remain poorly understood, but temporal associations between gestational food aversions and NVP suggest that the latter might, in part, condition the former [15, 20].

Gestational food aversions and NVP have been proposed as homeostatic mechanisms sheltering mother and fetus from harmful substances [14, 21–25]. This maternal and embryonic protection hypothesis proposes that gestational aversions and NVP serve as adaptive protective mechanisms. Changes in olfactory experience potentially contribute to increased NVP and food aversions during pregnancy. Although pregnancy does not alter absolute olfactory detection thresholds, it reduces odor identification accuracy and may increase perceived odor intensity, along with a slight decline in perceived odor pleasantness during the second trimester [6, 26]. These changes could be part of a protective adaptation [27]. Consistent with this idea, pregnant women often report unpleasant odors, including coffee, fried foods (a source of teratogenic acrylamide), cigarette smoke, and meat [28–30]. Moreover, adverse olfactory experience has been positively correlated with NVP [30, 31].

While NVP and disgust sensitivity share overlapping features, such as avoidance of pathogens, they are distinct processes. NVP is characterized by motility changes specific to pregnancy, whereas disgust sensitivity can be observed throughout life. Additionally, disgust sensitivity (but not NVP) may increase in response to broader environmental pathogenic threats [32]. Informed by a more complete understanding of immunological changes during pregnancy, Kaňková *et al*. (2022) [33] revised the compensatory prophylaxis hypothesis, suggesting that increases in disgust sensitivity during pregnancy may compensate for incomplete maternal success in balancing immune functioning. Of particular relevance, they found negative correlations between first-trimester disgust sensitivity and a mixture of pro- and anti-inflammatory cytokines (e.g. IFN-γ, IL-1β, IL-2, IL-4, IL-7, IL-17A, TNF-α, MCP-1, Eotaxin, RANTES, PDGF-BB, and FGF basic). They also found that NVP correlated negatively with certain cytokines (e.g. IL-1β, IL-2, IL-4, IL-9, IL-17A, TNF-α, MCP-1, Eotaxin, RANTES, G-CSF, and PDGF-BB). NVP has been linked to hormonal changes, particularly β-hCG and PAPP-A, which are essential for pregnancy progression and immune adaptation. Recent findings suggest opposing correlations between β-hCG and PAPP-A and both disgust sensitivity and NVP [34].

Building on previous research, we explore associations between changes in olfactory experience, food aversions, nausea and vomiting, and cytokines during early pregnancy. We examine both pro-inflammatory (Th1) and anti-inflammatory (Th2) cytokines, as their balance is fundamental for a successful pregnancy [35, 36]. We first explore correlations between odor and food aversions and NVP, then examine how each of these outcomes relates to cytokine levels**.** This allows us to investigate both the behavioral and biological immune systems [37], potentially illuminating how these two branches of immunity may work in tandem to protect mother and fetus.

## METHODS

### Study population

Our sample derives from Wave 2 of the Mothers’ Cultural Experiences (MCE) study [38–40]. Eligible participants, recruited between 2018 and 2020 at prenatal clinics in Southern California, were at least 18 years old, ≤17 weeks pregnant at enrollment, self-identified as Latina, Hispanic, Chicana, Mexicana, or Latin American, and spoke English or Spanish. In either language, participants provided informed, written consent after study procedures were described, then answered demographic questions (e.g. age, education, country of birth, and marital status) and questions on culture, identity, health, stressors, and relationships, followed by a morning blood draw. Fifty-nine individuals participated, but one did not complete the symptomatology questionnaire, resulting in a final sample of 58.

While the recruitment criteria are important for the MCE study’s overarching aims, the research questions addressed here are relevant to all pregnant cohorts. While acknowledging that not all pregnant individuals identify as women or become mothers, we use the terms “mother” and “woman” when discussing maternal biological activity, in contrast to fetal biological activity. This study was approved by the IRBs of all participating institutions with appropriate reliances. Procedures comply with the Declaration of Helsinki. Data are not publicly available because participants did not consent to sharing individual-level data.

### Symptomology assessment

Aversions, nausea, and vomiting were assessed with questionnaires administered at a second timepoint, at least 6 weeks after the blood draw during the focal pregnancy. Food aversions were assessed using validated questionnaires [15, 41], starting with “During your current pregnancy, have you experienced any aversions or a strong dislike towards a certain food or drink?” If participants answered yes, they were asked to specify toward which food(s) and drink(s) they had a strong dislike, among options such as fruit, fruit juice, sweet foods (chocolate, cookies, ice cream), tea, coffee, spicy or highly flavored food, meat, or other (specified as text). Similarly, odor aversions were assessed by asking, “During your current pregnancy, have you experienced any aversions or a strong dislike towards a certain smell?” Participants answering affirmatively were asked to specify toward which smell(s) they had a strong dislike, choosing from options such as tobacco smoke, coffee, colognes and perfumes, alcohol, body odor of individuals, or other (specified as text). For both questions, each response item was binary yes/no and participants were able to select multiple answers. Additionally, for descriptive purposes, we quantified the number of aversive foods or odors for each participant, treating this count as a continuous measure.

Nausea and vomiting were measured using validated questionnaires asking participants to recall the worst day they experienced nausea and vomiting during their current pregnancy and answer three questions [15, 42, 43]. Nausea duration was assessed by asking, “For how long did you feel nauseated or sick to your stomach that day?” with responses on a 5-point scale (not at all, less than 1 hour, 1–3 hours, 3–6 hours, more than 6 hours). Vomiting frequency was assessed with “How many times did you vomit or throw up that day?” with responses on a 4-point scale (never, 1–3 times, 4–7 times, more than 7 times). Retching frequency was assessed with “How many times did you have retching or dry heaves without bringing anything up that day?” using the same 4-point scale. We created a composite measure of nausea and vomiting by rescaling the answers for nausea, vomiting, and retching to a range of 0–1 and summing those values. A binary variable was created based on the composite measure using the median cut point.

### Cytokine measures

Cytokines were measured from non-fasting morning blood samples collected at 5–17 weeks’ gestation. Blood used for cytokine assays was collected by antecubital venipuncture into EDTA-treated vacutainers, kept at refrigerator temperature, and processed for plasma extraction within a few hours. Plasma aliquots were then frozen at −80°C until assayed. A multiplex assay was used to measure concentrations of the cytokines according to the manufacturer’s instructions (V-PLEX Proinflammatory Panel 1, Meso Scale Discovery, Meso Scale Diagnostics LLC, Rockville, MD). In some blood samples, cytokine levels that were below the detection limit were imputed using the minimum detectable levels for each cytokine: IL-1β (*n* = 1) at 0.04 pg/ml, IL-2 (*n* = 6) at 0.05 pg/ml, IL-12 (*n* = 26) at 0.05 pg/ml, IFN-γ (*n* = 1) at 0.15 pg/ml, TNF-α (*n* = 1) at TNF-α 0.10 pg/ml, IL-4 (*n* = 40) at 0.04 pg/ml, IL-10 (*n* = 1) at 0.03 pg/ml, IL-13 (*n* = 24) at 0.18 pg/ml. Cytokine composite scores were computed as the sum of the raw value of each cytokine: the Th1 composite included IL-1β, IL-2, IL-12, IFN-γ, and TNF-α; the Th2 composite included IL-4, IL-10, and IL-13. Prior to statistical analysis, composite scores and individual cytokine levels (e.g. IL-6 and IL-8) were natural log-transformed to reduce skew.

### Statistical analysis

In initial exploratory analyses, we used continuous variables for aversions and nausea and vomiting to understand the data’s natural patterns and associations. We conducted Kendall correlation tests to explore the associations among number of aversive odors, number of aversive foods, duration of nausea, frequency of vomiting, and frequency of retching. For the main multivariable regression analyses, we used binary variables to indicate the presence or absence of each aversion; and the degree to which nausea and vomiting were severe or not (severe: >median vs. not severe: ≤median). This was done to reduce the complexity of the model and minimize the risk of overfitting, especially considering our limited sample size. Using binary variables also renders the model more interpretable. We investigated the associations between cytokine levels and individual aversions and nausea and vomiting variables (odor aversion, nausea and vomiting, and food aversion). We used two models with progressive adjustment for potential confounding factors identified from directed acyclic graphs based on prior research and data availability. Model 1 represented a crude model. Model 2 adjusted for gestational age at blood draw (weeks), maternal acute or chronic condition (present vs. none; see Supplemental Note for details), smoking history before pregnancy (yes vs. no), and parity. Given our small sample size, a propensity score was employed in Model 2 to control for these covariates effectively. Analyses were performed using R, version 4.2.2.

As sensitivity analyses, we examined whether the most commonly observed aversions were linked to cytokine levels, to explore if specific types of aversions (such those toward certain odors or foods) had distinct immunomodulatory profiles. Additionally, we conducted analyses on IL-6 and IL-8, which are not included in the Th1 and Th2 composites yet play critical roles in pregnancy [44]. In the primary analyses, more than half of IL-4 levels were undetectable, hence values were imputed using the minimum detectable levels. To examine whether this might bias the results, we repeated the analyses by using an alternate Th2 composite and Th1:Th2 ratio without these imputations. Finally, to facilitate comparison between our results and those of Kaňková *et al*. (2022) [33], we created a Th1:Th2 composite ratio using those cytokines measured in both studies (IL-1β, IL-2, IL-4, and TNF-α).

## RESULTS

**Figure 2:**
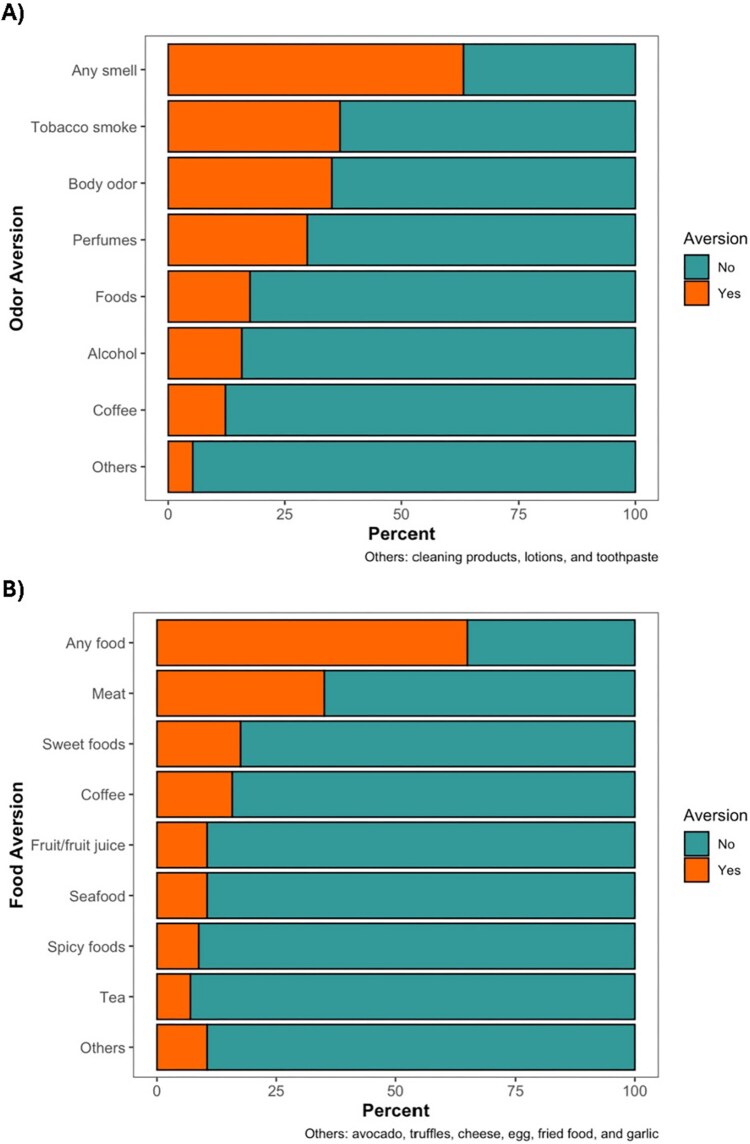
Percentage of (A) odor and (B) food aversions reported during pregnancy by our analytic cohort.

In our sample of 58 women, aged 20–42 (Table 1), a majority were born outside the USA (59%), had education levels equivalent to graduating high school (72%), and were employed (55%). Most were married (55%) and cohabitating with the baby’s father (83%). Gestational ages at blood draw ranged from 5 to 17 weeks, within the first and second trimesters. The mean prepregnancy BMI was 30 kg/m^2^ (SD = 6), indicating that most were overweight before becoming pregnant. About 16% were food insecure. Odor aversions were reported by about 64%, most frequently toward tobacco smoke (Fig. 2). Food averasions were also reported by about 64%, often toward meat. Nausea was reported by 67%, and vomiting by 66%.

**Table 1:** Characteristics of study population.

	Total (*n* = 58)
Number of aversive odors	
Mean (SD)	1.6 (1.6)
Median [min, max]	1 [0, 5]
Number of aversive foods	
Mean (SD)	1.2 (1.2)
Median [min, max]	1 [0, 5]
Duration of nausea (hours)	
Mean (SD)	3.7 (3.2)
Median [min, max]	2 [0, 8]
Frequency of vomiting (times)	
Mean (SD)	2.3 (2.5)
Median [min, max]	2 [0, 9]
Frequency of retching (times)	
Mean (SD)	1.8 (2.5)
Median [min, max]	2 [0, 9]
Maternal age (years)	
Mean (SD)	31 (5.7)
Median [min, max]	30.3 [20.1, 42]
Country born, *n* (%)	
USA	24 (41.4)
Mexico	23 (39.7)
El Salvador and Guatemala	11 (19.0)
Education, *n* (%)	
Less than high school	8 (13.8)
High school or equivalent	42 (72.4)
Any college or beyond	8 (13.8)
Work status, *n* (%)	
Employed	32 (57.1)
Unemployed	24 (42.9)
Missing	2
Marital status, *n* (%)	
Single	26 (44.8)
Married	32 (55.2)
Cohabitating with baby’s father, *n* (%)	
No	10 (17.2)
Yes	48 (82.8)
History of smoking before pregnancy, *n* (%)	
No	50 (86.2)
Yes	8 (13.8)
Alcohol consumption during pregnancy, *n* (%)	
No	52 (89.7)
Yes	6 (10.3)
Gestational age at blood draw (weeks)	
Mean (SD)	12.4 (2.9)
Median [min, max]	12.6 [5.1, 17.6]
Gestational age at questionnaire administration (weeks)	
Mean (SD)	24.5 (3.17)
Median [min, max]	24.4 [16.6, 33.1]
Parity	
Mean (SD)	1.3 (1.4)
Median [min, max]	1 [0, 6]
Gravity	
Mean (SD)	3.4 (2.8)
Median [min, max]	3 [1, 14]
Primiparas, *n* (%)	
No	44 (77.2)
Yes	13 (22.8)
Missing	1
Prepregnancy BMI	
Mean (SD)	29.5 (5.8)
Median [min, max]	28.9 [18, 49.1]
Food insecure, *n* (%)	
No	49 (84.5)
Yes	9 (15.5)

**Figure 3:**
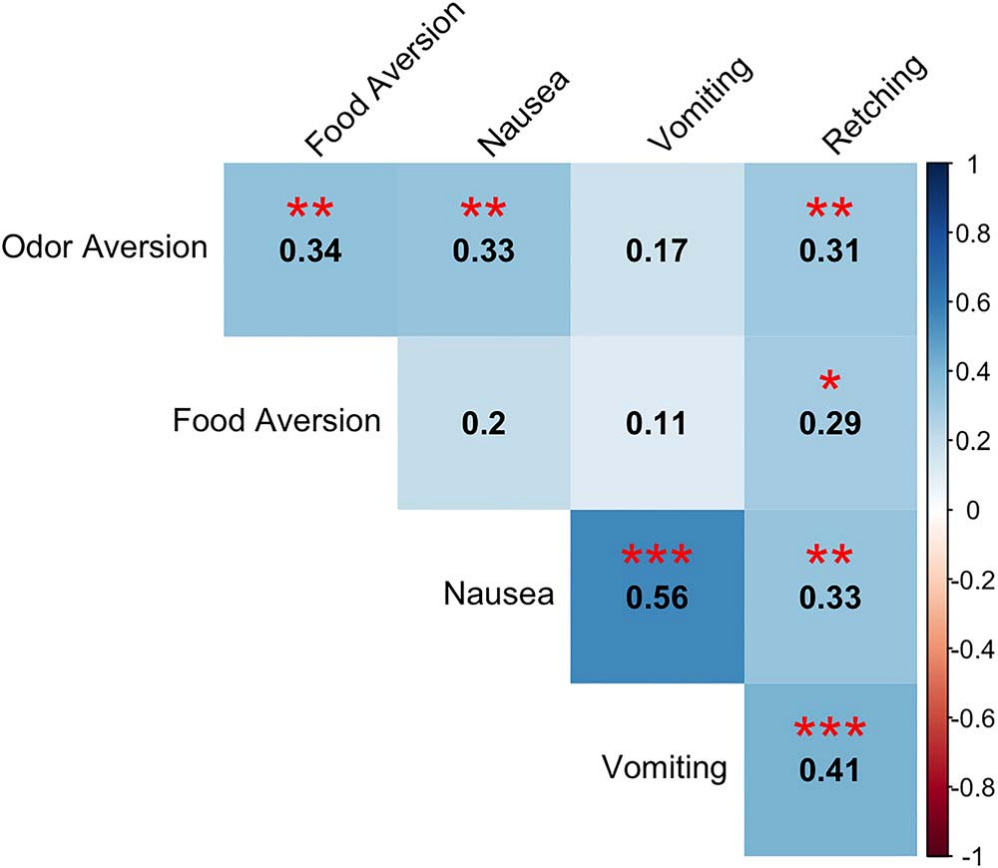
Kendall correlation coefficients were calculated to assess the corrections between number of aversive odors, number of aversive foods, duration of nausea, frequency of vomiting, and frequency of retching. Significance levels are denoted as follows: ^***^P < .001, ^**^P < .01, ^*^P < .05. Positive correlations are depicted in blue, as represented by the right-most scale in the figure. In this figure, all correlations were positive, so depth of color reflects the strength of the correlation.

The number of aversive odors correlated positively with nausea duration (*τ*_b_ = 0.33, *P* = .002), frequency of retching (*τ*_b_ = 0.31, *P* = .006), and the number of aversive foods (*τ*_b_ = 0.34, *P* = .002), i.e. individuals with more odor aversions experienced longer-lasting nausea, more frequent retching, and more food aversions (Fig. 3). Nausea duration correlated positively with frequency of vomiting (*τ*_b_ = 0.56, *P* < .001) and retching (*τ*_b_ = 0.33, *P* = .003), suggesting that, as would be expected, prolonged nausea was associated with more frequent episodes of vomiting and retching. Retching frequency and the number of aversive foods were moderately positively correlated (*τ*_b_ = 0.29; *P* = .012).

In fully adjusted multivariable linear regression analyses, odor aversion was positively associated with the Th1 composite cytokine level, with an estimate of 0.30 (95% CI = −0.05, 0.65; Table 2), implying that pregnant women with odor aversions may have higher pro-inflammatory cytokine levels compared to those without odor aversions. Participants who reported an aversion to tobacco smoke appeared to exhibit a shift toward Th1 immune responses with a lower Th2 composite cytokine level (*β* = −0.17; 95% CI = −0.30, −0.03; Table S1) and a higher Th1:Th2 composite ratio (*β* = 0.20; 95% CI = 0.03, 0.37) compared with those without such an aversion during pregnancy.

**Table 2:** Association of odor aversion, nausea, and food aversion with cytokines.

	Estimate (95% CI)	
	Model 1^a^	Model 2^b^
Odor aversion		
Th1 composite	0.23 (−0.07, 0.54)	0.30 (−0.05, 0.65)
Th2 composite	−0.07 (−0.20, 0.06)	−0.05 (−0.20, 0.10)
Th1:Th2 composite	0.10 (−0.06, 0.27)	0.15 (−0.04, 0.33)
Nausea and vomiting		
Th1 composite	0.04 (−0.26, 0.34)	0.01 (−0.30, 0.32)
Th2 composite	−0.10 (−0.22, 0.03)	−0.10 (−0.23, 0.03)
Th1:Th2 composite	0.11 (−0.04, 0.27)	0.14 (−0.02, 0.30)
Food aversion		
Th1 composite	0.36 (0.06, 0.66)	0.40 (0.05, 0.74)
Th2 composite	0.06 (−0.07, 0.19)	0.05 (−0.10, 0.20)
Th1:Th2 composite	0.10 (−0.06, 0.27)	0.10 (−0.09, 0.30)

a Model 1 represented a crude model.

b Model 2 adjusted for gestational age at blood draw, maternal acute or chronic condition, smoking history before pregnancy, and parity.

Nausea and vomiting were positively associated with the Th1:Th2 composite ratio (*β* = 0.14; 95% CI = −0.02, 0.30), suggesting that pregnant women experiencing severe nausea and vomiting might have a higher proportion of pro-inflammatory cytokines relative to anti-inflammatory cytokines compared to those not experiencing severe nausea and vomiting. Similar trends appeared for the alternative Th1:Th2 composite ratios (Table S2). As expected, the exclusion of IL-4 imputation resulted in a stronger effect (*β* = 0.26; 95% CI = −0.04, 0.55) due to a smaller Th2 composite denominator. Thus, using imputed IL-4 values provides a more balanced representation of the Th2 composite, ensuring reliable comparison, and avoiding overestimation of the Th1:Th2 composite ratio. Additionally, we recalculated the Th1:Th2 ratio using those cytokines measured in both our study and Kaňková *et al.* (2022) [33] (IL-1β, IL-2, IL-4, and TNF-α). Unlike their report of negative associations between NVP and both individual pro-inflammatory (IL-1β, IL-2, TNF-α) and anti-inflammatory (IL-4) cytokines, our recalculated Th1:Th2 composite ratio was positively associated with NVP (*β* = 0.30; 95% CI = −0.47, 1.07). Hence, reanalyzing our data using only the cytokines measured in Kaňková *et al*.’s study produced results consistent with our primary findings, indicating a shift toward a pro-inflammatory Th1 immune response in severe NVP.

Food aversions were positively associated with the Th1 cytokine composite (*β* = 0.40; 95% CI = 0.05, 0.74), indicating that participants reporting food aversions tend to have higher pro-inflammatory cytokine levels compared to those without food aversions. When focusing only on meat aversions, we also found an increase in the Th1 cytokine composite (*β* = 0.17; 95% CI = −0.17, 0.05; Table S1).

## DISCUSSION

Our study contributes to the nascent exploration of the interplay between the maternal immune system’s inflammatory responses and pregnancy-related aversions, nausea, and vomiting. By integrating analyses of odor and food aversions with nausea and vomiting in pregnancy, our findings illuminate their complex relationships with immune function. This investigation extends beyond the commonly explored sensory and behavioral dimensions of pregnancy-related aversions, illuminating their underpinnings in early pregnancy’s inflammatory response.

We found that experiencing stronger negative reactions to odors was associated with more severe nausea/vomiting and greater food aversions in early pregnancy. This pattern may reflect a single adaptive mechanism driving all three symptoms, or a sequence where odor sensitivity triggers nausea/vomiting, which then leads to food aversions. We also observed that individuals who experience severe nausea and vomiting may develop strong food aversions. These positive correlations afford several possible explanations. First, changes in the hedonic value of olfactory stimuli, the onset of targeted food aversions, and increases in nausea and vomiting may all be underpinned by a single adaptive mechanism. Alternatively, these events may occur sequentially, with changes in olfactory responses leading to nausea and vomiting, which in turn contribute to learned food aversions [45]. Although our data lack the detail and temporal resolution to adjudicate between these possibilities, the short-lived nature of gestational food aversions [46] suggests they may not align with classical conditioned food aversions, which typically endure longer [47]. Nevertheless, we recognize that the coordinated nature of these symptoms does not definitively indicate adaptive mechanisms, as they could be nonadaptive consequences of immune changes.

Our study evaluates the biological underpinning of these phenomena, revealing a pattern of higher pro-inflammatory cytokines and lower anti-inflammatory cytokines, leading to a higher Th1:Th2 ratio, among pregnant women who experience odor aversions, food aversions, and higher levels of nausea and vomiting. This contrasts with Kaňková *et al*. (2022) [33], who reported negative associations between NVP and individual Th1 and Th2 cytokines in the first trimester. When we recalculated the Th1:Th2 ratio using only cytokines measured in both studies, we found positive associations between symptoms and a shift toward Th1 responses, similar to our primary findings. However, methodological differences, including Kaňková *et al*.’s use of individual cytokines versus our composite approach, complicate direct comparisons. Additionally, Kaňková *et al*. employed orthogonal partial least squares models, which may overfit the data, potentially explaining some of the contrast between their findings and ours.

Coupled with previous studies suggesting that the absence of NVP may be associated with poorer outcomes [48, 49], our findings imply that women who do not experience these symptoms may have different immune profiles. If elevated Th1:Th2 ratio indicate a robust immune response supporting implantation and placentation, the absence of such an elevation might pose a risk for unsuccessful implantation. Future studies should clarify the relationship between Th1:Th2 ratios and pregnancy outcomes to determine whether this ratio can serve as an early marker of pregnancy health.

Our study is subject to several limitations. Our sample is relatively small and represents a narrow demographic group (Latina women aged 20–42), which may limit generalizability and reduce the power to detect smaller but clinically relevant associations. However, asking this research question in a single demographic group may reduce certain sources of confounding. While there is much cultural diversity among Latinas living in the USA, there is also substantial overlap in the cultural and religious beliefs as well as taboos about foods during pregnancy, which could influence aversions and NVP. We employed self-report measures for aversions and nausea, which may be subject to recall bias, particularly given the time elapsed between symptom onset and questionnaire completion; conversely, the inclusion of individuals in the second and early third trimesters may lead to underestimate prevalence and intensity of aversions, nausea, and vomiting, as participants’ responses may have been biased toward their most recent experiences since these symptoms typically peak during the first trimester. Future research with larger and more diverse samples is needed, along with development of more refined questionnaires to capture a broader range of symptom severity. Our measurement of cytokines from a single blood draw may be more representative for cytokines that exhibit stability across the first half of pregnancy, such as IL-1β, IL-8, IL-12, TNF-α, IL-4, and IL-13, which were shown to have median intra-individual changes of less than 5% from 10 to 18 weeks' gestation, and less representative for IL-2, IL-6, and IL-10, which exhibited declines ranging 15.7–20.8% over the same period [50]. Longitudinal studies that track the full range of these variables throughout pregnancy will offer a more comprehensive understanding of their relationships and allow for the investigation of the temporal order in which symptoms and immune changes manifest.

Although few participants in our study had chronic conditions such as arthritis, autoimmune disorders, or depression, these conditions can affect cytokine levels and immune function. Human chorionic gonadotropin (hCG), an immunomodulating hormone that promotes tolerance of the embryo, may influence both NVP and cytokine levels. Although hCG is typically associated with anti-inflammatory effects at the maternal-fetal interface, it may exert pro-inflammatory effects in maternal circulation, such as increasing plasma concentrations of IL-1β [51]. The relationship between NVP, hCG, and cytokine levels thus warrants further investigation. Furthermore, we measured neither sex steroid hormones nor growth differentiation factor 15 [19], yet their interplay with inflammation, nausea, vomiting, and aversions during pregnancy may prove important.

Various psychological and behavioral pathways should be examined in larger studies powered to test causal mediation. Depression has been positively associated with peripheral inflammation in nonpregnant populations, though associations are inconsistent in pregnant women [52]. Stress and depression have been positively associated with NVP during pregnancy [53]. Negative affectivity and inflammation (which seem to be reciprocally causal; [54]) may contribute to NVP. Likewise, a psychological adaptation for fetal protection may be triggered by inflammation and manifest as a combination of negative affect, food and smell aversions, and NVP.

Food insecurity may also influence aversions, nausea and vomiting, and immune function during pregnancy by acting as both a mediator and moderator. As a mediator, it contributes to nutritional deficiencies, chronic stress, and elevated cortisol levels, all of which can exacerbate NVP and aversions and increase systemic inflammation [55–57]. As a moderator, limited food access may override aversions, forcing consumption of non-preferred foods [58], a behavior known as pica. Our previous study on pica also found elevated cortisol levels among pregnant women practicing pica [59], suggesting a potential link between food insecurity, stress response, and immune dysregulation. While only a small number of participants in our study reported food insecurity, future research should explore its role in pregnancy-related immune changes and symptom severity.

We observed associations between odor and food aversions, nausea and vomiting, and the maternal immunomodulatory environment during pregnancy. While evidence from previous studies bearing on the hypothesis that morning sickness as an adaptive mechanism is mixed [19, 60, 61], our findings support its potential role in protecting fetal development. These findings extend the understanding of pregnancy-related aversions and nausea by exploring their potential immunomodulatory association, suggesting that aversions and nausea, alongside first-trimester pregnancy-related experiences and the immune system’s pro-inflammatory shift, may help balance between defense against infections and fetal adaptation. Future research should evaluate the immune profiles of mothers without these symptoms and their impact on pregnancy outcomes.

## Supplementary Material

Kwon_aversion_suppl_R1_20250427_eoaf016

## Data Availability

Data are not publicly available because participants did not consent to sharing individual-level data publicly.
