# Supplementary material for: Of scents and cytokines: How olfactory and food aversions relate to nausea and immunomodulation in early pregnancy
Source: Evol Med Public Health. 2025 Sep 24;13(1):269–80. doi: 10.1093/emph/eoaf016 (PMC12476167; doi:10.1093/emph/eoaf016)
Supplement: Kwon_aversion_suppl_R1_20250427_eoaf016 [file kwon_aversion_suppl_r1_20250427_eoaf016.docx]

**SUPPLEMENTAL INFORMATION
Table S1.** Association between most commonly observed aversions and cytokines. Blue cells indicate positive associations and red cells indicate negative associations. The intensity of the color corresponds to the effect size, with darker shades representing larger absolute values.

|  | **Estimate (95% CI)** | |
| --- | --- | --- |
|  | **Model 1**^a^ | **Model 2**^b^ |
| **Odor: Tobacco Smoke Aversion** | | |
| Th1 composite | 0.08 (-0.23, 0.39) | 0.04 (-0.29, 0.37) |
| Th2 composite | -0.15 (-0.28, -0.03) | -0.17 (-0.30, -0.03) |
| Th1:Th2 composite | 0.16 (-0.00, 0.32) | 0.20 (0.03, 0.37) |
| IL-6 | -0.12 (-0.29, 0.04) | -0.15 (-0.32, 0.02) |
| IL-8 | -0.04 (-0.22, 0.14) | -0.00 (-0.19, 0.19) |
| **Food: Meat Aversion** |  |  |
| Th1 composite | 0.14 (-0.17, 0.45) | 0.17 (-0.17, 0.50) |
| Th2 composite | 0.00 (-0.13, 0.14) | -0.02 (-0.16, 0.12) |
| Th1:Th2 composite | 0.01 (-0.16, 0.17) | -0.01 (-0.19, 0.17) |
| IL-6 | -0.00 (-0.17, 0.17) | 0.02 (-0.16, 0.20) |
| IL-8 | 0.04 (-0.15, 0.22) | 0.04 (-0.16, 0.24) |

^a­­^Model 1 represented a crude model.

^b^Model 2 adjusted for gestational age, maternal acute or chronic condition, smoking history before pregnancy, and parity.

**Table S2.** Association of odor aversion, nausea, and food aversion with alternate Th2 composite, individual cytokines, and cytokine composite^a^ derived from Kaňková et al. (2022). Blue cells indicate positive associations and red cells indicate negative associations. The intensity of the color corresponds to the effect size, with darker shades representing larger absolute values.

|  | **Estimate (95% CI)** | |
| --- | --- | --- |
|  | **Model 1**^b^ | **Model 2**^c^ |
| **Odor Aversion** |  |  |
| Alternate Th2 composite | -0.08 (-0.21, 0.06) | -0.05 (-0.20, 0.11) |
| Alternate Th1:Th2 composite | 0.25 (-0.04, 0.55) | 0.24 (-0.10, 0.58) |
| Kaňková Th1:Th2 composite | -0.16 (-0.89, 0.58) | -0.20 (-0.96, 0.57) |
| IL-6 | -0.06 (-0.23, 0.11) | -0.07 (-0.26, 0.13) |
| IL-8 | -0.00 (-0.19, 0.18) | 0.04 (-0.18, 0.25) |
| **Nausea and Vomiting** |  |  |
| Alternate Th2 composite | -0.10 (-0.23, 0.03) | -0.10 (-0.24, 0.04) |
| Alternate Th1:Th2 composite | 0.27 (-0.02, 0.55) | 0.26 (-0.04, 0.55) |
| Kaňková Th1:Th2 composite | 0.27 (-0.46, 1.00) | 0.30 (-0.47, 1.07) |
| IL-6 | 0.06 (-0.10, 0.22) | 0.04 (-0.13, 0.20) |
| IL-8 | -0.00 (-0.18, 0.18) | 0.03 (-0.15, 0.21) |
| **Food Aversion** |  |  |
| Alternate Th2 composite | 0.06 (-0.08, 0.20) | 0.05 (-0.11, 0.21) |
| Alternate Th1:Th2 composite | 0.13 (-0.17, 0.44) | 0.24 (-0.11, 0.58) |
| Kaňková Th1:Th2 composite | -0.02 (-0.84, 0.79) | -0.01 (-1.14, 1.12) |
| IL-6 | -0.03 (-0.19, 0.14) | -0.02 (-0.21, 0.17) |
| IL-8 | 0.01 (-0.18, 0.19) | 0.00 (-0.22, 0.22) |

^a^Kaňková composite included IL-1β, IL-2, IL-4, IL-17A, and TNF-α; note that Kaňková et al. did not measure odor or food aversions, hence said model output is presented here for illustrative rather than comparative purposes.

^b^Model 1 represented a crude model.

^c^Model 2 adjusted for gestational age, maternal acute or chronic condition, smoking history before pregnancy, and parity.

**SUPPLEMENTAL NOTE**

For the covariate maternal acute or chronic condition, the following conditions are included under acute or chronic conditions: arthritis (n = 2), asthma (n = 1), autoimmune disorder (n = 1), carcinoid tumor (n = 1), chronic low back pain (n = 1), depression (n = 1), diabetes (type 1 or type 2; n = 4), epilepsy (n = 1), hemiplegic migraines (n = 1), high blood pressure (n = 1), kidney transplant (n = 1), stroke (n = 1), tachycardia (n = 1), and xerosis (n = 1).
